# Pyridoxine dipharmacophore derivatives as potent glucokinase activators for the treatment of type 2 diabetes mellitus

**DOI:** 10.1038/s41598-017-16405-2

**Published:** 2017-11-22

**Authors:** Mikhail S. Dzyurkevich, Denis A. Babkov, Nikita V. Shtyrlin, Olga Yu. Mayka, Alfiya G. Iksanova, Pavel M. Vassiliev, Konstantin V. Balakin, Alexander A. Spasov, Vadim V. Tarasov, George Barreto, Yurii G. Shtyrlin, Gjumrakch Aliev

**Affiliations:** 1 Kazan (Volga region) Federal University, Kremlyovskaya 18, Kazan, 420008 Russia; 2 Volgograd State Medical University, Pavshikh Bortsov Sq. 1, Volgograd, 400131 Russia; 3 I.M. Sechenov First Moscow State Medical University, Trubetskaya St. 8, bld 2, Moscow, 119991 Russia; 4 Institute of Pharmacy and Translational Medicine, Sechenov First Moscow State Medical University, 119991 Moscow, Russia; 5 Departamento de Nutrición y Bioquímica, Facultad de Ciencias, Pontificia Universidad Javeriana, Bogotá D.C., Colombia; 6 Instituto de Ciencias Biomédicas, Universidad Autónoma de Chile, Santiago, Chile; 7 GALLY International Biomedical Research & Consulting LLC 7733 Louis Pasteur Dr. Suite #328, San Antonio, TX 78229 USA; 8 School of Health Science and Healthcare Administration, University of Atlanta, E. Johns Crossing, #175, Johns Creek, GA 30097 USA; 9 Institute of Physiologically Active Compounds Russian Academy of Sciences, Chernogolovka, 142432 Russia

## Abstract

Glucokinase is one of the promising targets for glucose-lowering agents, and the development of GK activators are now considered as one of the most promising strategies for the treatment of type 2 diabetes mellitus. In this work, a series of novel symmetric molecular constructs, in which two pyridoxine moieties are connected via sulfur-containing linkers, have been synthesized and tested *in vitro* for glucokinase activation potential. The enzyme activation rates by two most active compounds at 100 μM (~150% and 130%) were comparable to that of the reference agent PF-04937319 (~154%). Both leading compounds demonstrated low cytotoxicity and excellent safety profile in acute toxicity experiment in rats after oral administration with LD_50_ exceeding 2000 mg/kg of body weight. Binding mode of the active compounds in comparison with the reference agent was studied using molecular docking. The leading compounds represent viable preclinical candidates for the treatment of type 2 diabetes mellitus, as well as a promising starting point for the design of structural analogs with improved activity.

## Introduction

Diabetes mellitus is a widespread and serious chronic disease, which has become an issue not only in developed regions of the world, but also in low- and middle-income countries over the past decade. The global prevalence of diabetes has nearly doubled since 1980, rising from 4.7% to 8.5%^1^.

The main goal in treatment of diabetes is the control of the blood glucose level. This goal can be achieved with the injections of insulin in case of type 1 diabetes. However, the treatment of type 2 diabetes mellitus (T2DM) is more complicated due to insulin resistance. The following types of drugs are used in the current clinical practice for the treatment of T2DM: insulin release increasing drugs (sulfonyl ureas), biguanides, in which the mechanism of action is not clear yet, nuclear peroxisome proliferator-activated receptor-γ agonists (e.g., thiazolidinediones), α-glucosidase inhibitors, dipeptidyl peptidase-4 inhibitors, insulin secretagogues (injectable incretin analogues), and sodium/glucose cotransporter 2 inhibitors^2^. If not controlled, diabetes may be associated to cognitive dysfunction, dementia and systemic metabolic syndrome^3–5^.

Glucokinase (GK) or hexokinase IV/D is one of the promising targets for glucose-lowering agents. This enzyme facilitates the conversion of glucose to glucose-6-phosphate that is the first step both in glycolysis and glycogen synthesis. GK has molecular weight of 50 kDa and is mainly expressed in liver and pancreas^6^. GK activity can be enhanced by small molecules binding to its allosteric site^7^, and the development of GK activators (GKAs) is now considered as one of the most promising strategies for T2DM treatment. Several GKAs reached phase II clinical trials. Among them are **Piragliatin** ^8^, **AMG-151** (**ARRY-403**)^9^, **PF-04937319** ^10^, and **RO-5305552** (**HMS5552**)^11^ shown in Suppl. Fig. 1. However, despite extensive studies in this area for the last 15 years, there are currently no GK activators that reached the market. The known GK activators have a number of side effects, such as narrow therapeutic window and concerns regarding potential effects on lipid metabolism, which strongly limit their potential utility and explain the reasons for failure in clinical trials. Therefore, the design and discovery of novel chemotypes of GKAs which do not possess such disadvantages is of a great interest for the pharmaceutical industry^12^.

In the framework of our GKAs discovery strategy based on screening of diverse chemical libraries, we have performed a virtual screening procedure using a molecular docking approach. The obtained *in silico* hits were then screened in *in vitro* assays. As a result, we have obtained a series of active compounds reported in this paper. These molecules appeared to be a novel chemotype of GKAs, which have not been described to-date in the scientific literature in the field.

## Results

**Figure 1:**
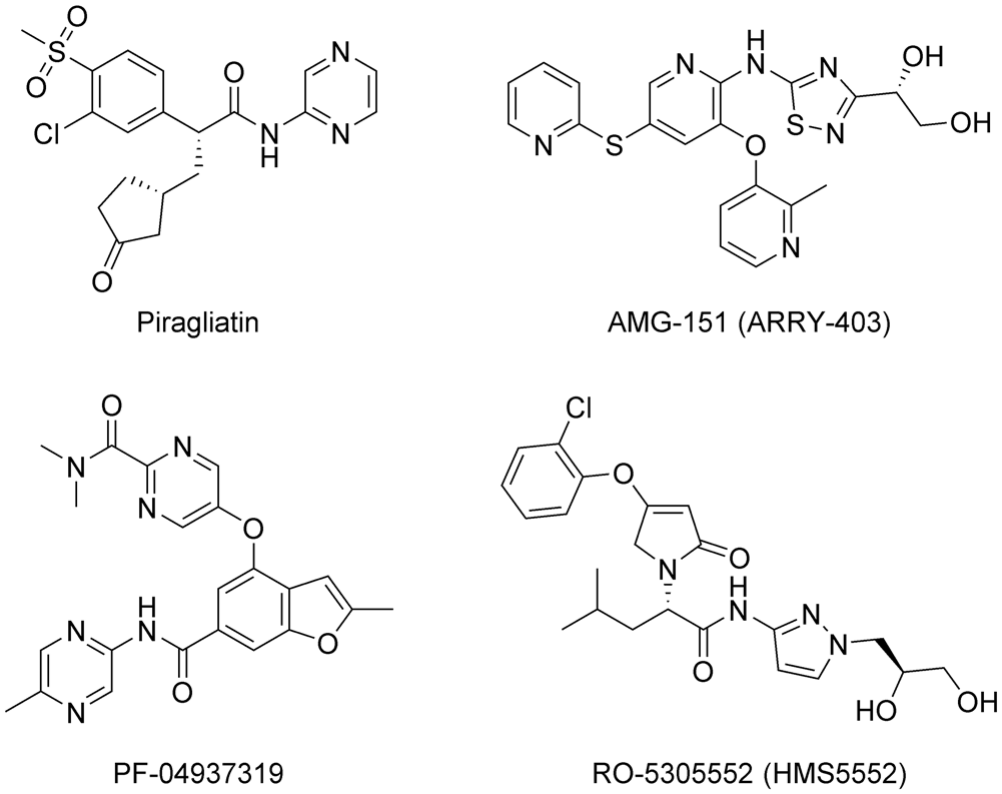
Synthetic route of compounds used. (**a**) Na_2_S_x_, TBAB, H_2_O-CHCl_3_, rt, 12 h; (**b**) H_2_O, HCl, 40 °C, 3 h; (**c**) H_2_O_2_, AcOH/H_2_O, rt, 4 h; (**d**) H_2_O_2_, AcOH/H_2_O, 50 °C, 4 h.

In this paper, we present a new chemical type of GKAs. The synthetic route is shown in Fig. 1. Chloride **2** was synthesized from initial pyridoxine hydrochloride **1** using our previously reported methods^13–15^. Interaction of **2** with Na_2_S or Na_2_S_2_ in the presence of catalytic amounts of tert-butyl ammonium bromide (TBAB) in a heterophase H_2_O-CHCl_3_ medium led to dimers **3** and **4**, respectively. These compounds were then hydrolyzed under mild acidic conditions to give the corresponding hexaols **5** and **6**. Oxidation of sulfide **5** with 2-fold excess of H_2_O_2_ in AcOH/H_2_O under room temperature, or 8-fold excess of H_2_O_2_ in the same solvent mixture at 50 °C led to sulfoxide **7** of sulfone **8**, respectively. The synthesis is straightforward and reproducible.

The ability of the synthesized compounds **5**–**8** to activate GK *in vitro* was then studied. **PF-04937319** ^10^ (Suppl. Fig. 1), a potent GK activator that had been developed in phase II clinical studies at Pfizer for the oral treatment of type 2 diabetes (discontinued in 2015), was used as a positive control. All the studied compounds appeared to be potent GK activators (Table 1). The nature of the linking sulfur-containing group significantly influenced the activity level. Thus, the GK activation rates by compounds **5** and **6** at 100 μM (~150% and 130%, respectively) were comparable to that of the reference drug (~154%) at the same concentration. Compounds **7** and **8** were less active (the activation rates 84.5% and 90.7%, respectively) under the same conditions. The 50% effective concentrations of the leading compounds **5** and **6** (18.6 and 33.4 μM) were approx. 3–5 times higher than EC_50_ of **PF-04937319**. It is interesting and practically important to understand possible reasons for the increased activity of sulfide **5** in comparison with its oxidized derivatives **7** and **8**. Analysis of ^1^H NMR data reveals strong intramolecular interactions between the linker SO (or SO_2_) groups and the hydroxymethyl groups in position 5 of the pyridoxine ring. Thus, a splitted signal is observed corresponding to H_a_ and H_b_ protons of the 5-hydroxymethyl group in ^1^H NMR spectrum of sulfoxide **7**; at the same time, such a splitting is not found in ^1^H NMR spectrum of sulfide **5**. This effect can be explained by a hindered rotation of the 5-hydroxymethyl group around the C_Ar_–C(OH) bond that can stabilize molecular conformations which are not optimal for effective binding.

**Table 1:** Effect of studied compounds on GK activity and the docking energies of new compounds in the GK allosteric site.

**Compound**	**GK activating properties**		
	**% of activation at 100** **μM (m ± SEM)**	**EC**_**50**_, **μM (95% C.I.)**	ΔE, kcal/mol*
5	150.6 ± 1.8	18.6 (12.1–24.5)	−7.8
6	130.5 ± 1.0	33.4 (30.6–39.5)	−7.9
7	84.5 ± 1.3	43.8 (40.2–48.7)	−8.3
8	90.7 ± 1.2	47.1 (41.4–49.4)	−8.3
PF-04937319	154.4 ± 5.3	6.80 (2.96–9.61)	−10.7

Cytotoxicity of compound **5–8** was evaluated in human skin fibroblast (HSF) cells. All the compounds demonstrated low cytotoxicity with CC_50_ > 1.0 mg/mL. Acute toxicity of the leading compounds **5** and **6** was estimated in rats following oral administration. It was found that LD_50_ for both compounds exceeded 2000 mg/kg of body weight (Table 2). The obtained *in vitro* cytotoxicity data well correlate with the observed low acute toxicity in animals, thus suggesting very good potential safety of the obtained compounds.

**Table 2:** Acute toxicity of compounds **5** and **6**.

**Compound**	**5**	**6**
	**LD**_**50**_ **, mg/kg of body weight**	
Rats/female (n = 6)	>2000	>2000
Rats/male (n = 6)	>2000	>2000

All the most powerful GKAs interact with the allosteric site, which is confirmed by the method of point mutations^16^. The binding mode of the obtained compounds **5**–**8** and the reference agent **PF-04937319** to the allosteric site of GK was analyzed using molecular docking approach according to previously described method^17^. The values of the minimum docking energy of the five compounds are given in Table 1. Taking into account the error of the method, ΔE values of the new compounds **5**–**8** do not significantly differ from each other, while ΔE value of the reference agent is substantially lower thus suggesting its higher activity. Thus, the calculated energy values are symbatic with the observed EC50 levels (Table 1), all points are within 95% confidence interval. Probably, the level of activity of compounds 5–8 is determined not only by the energy, but also by the nature of the binding and the poses of the molecules into the GK site.

**Figure 2:**
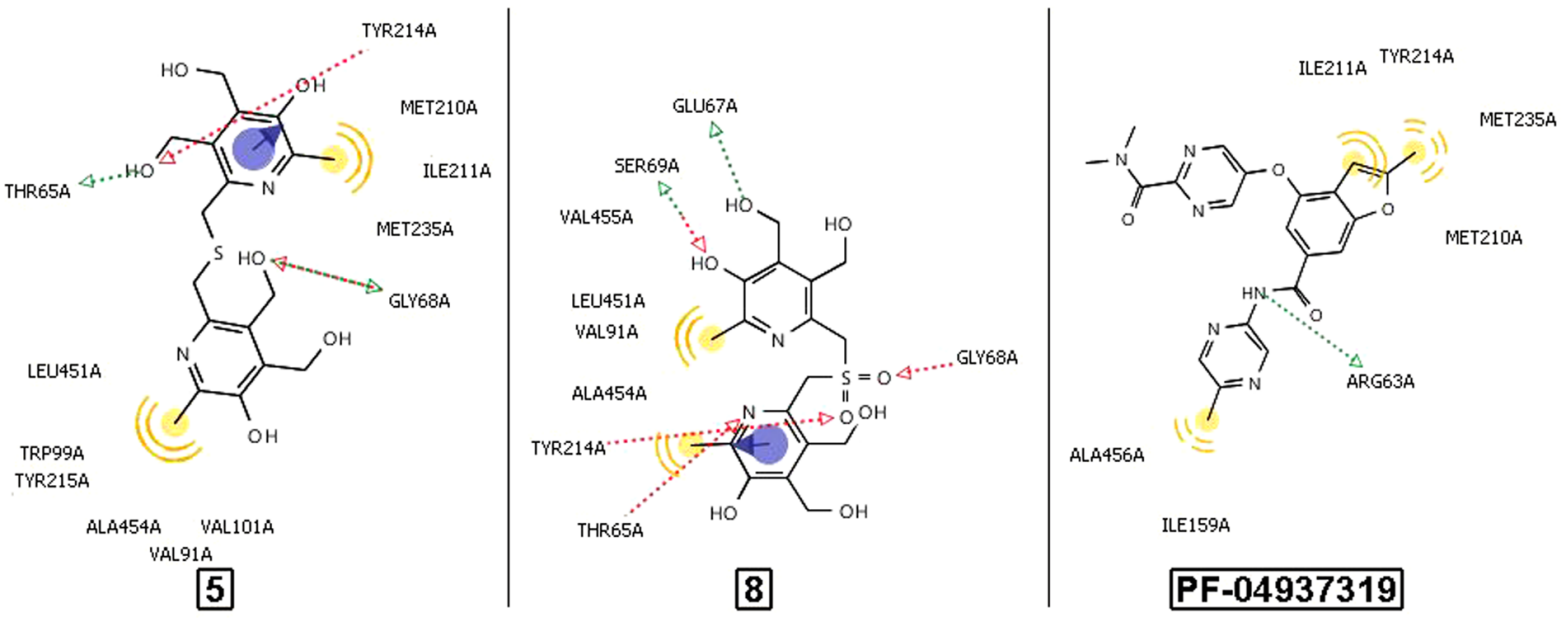
The key binding interactions of compounds **5**, **8** and **PF-04937139** in the allosteric site of GK.

**Figure 3:**
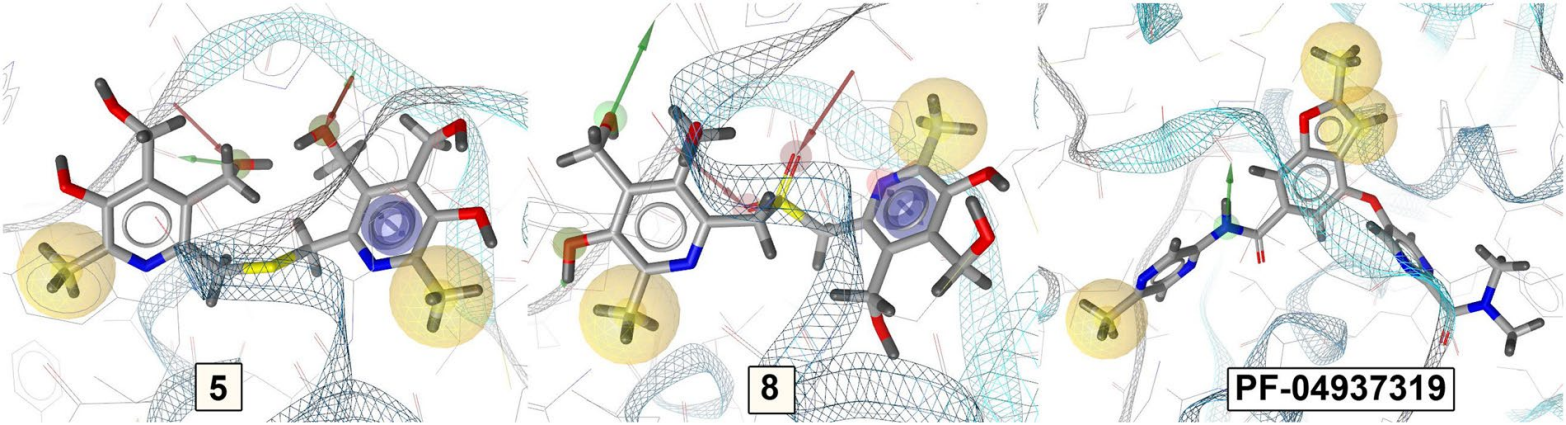
The docking poses of the studied compounds in the GK site.

The key binding interactions of the most active compound **5**, the least active compound **8**, and the reference agent **PF-04937139** in the allosteric site of GK are shown in Fig. 2. The docking poses of all three compounds in the GK site are shown in Fig. 3.

**Figure 4:**
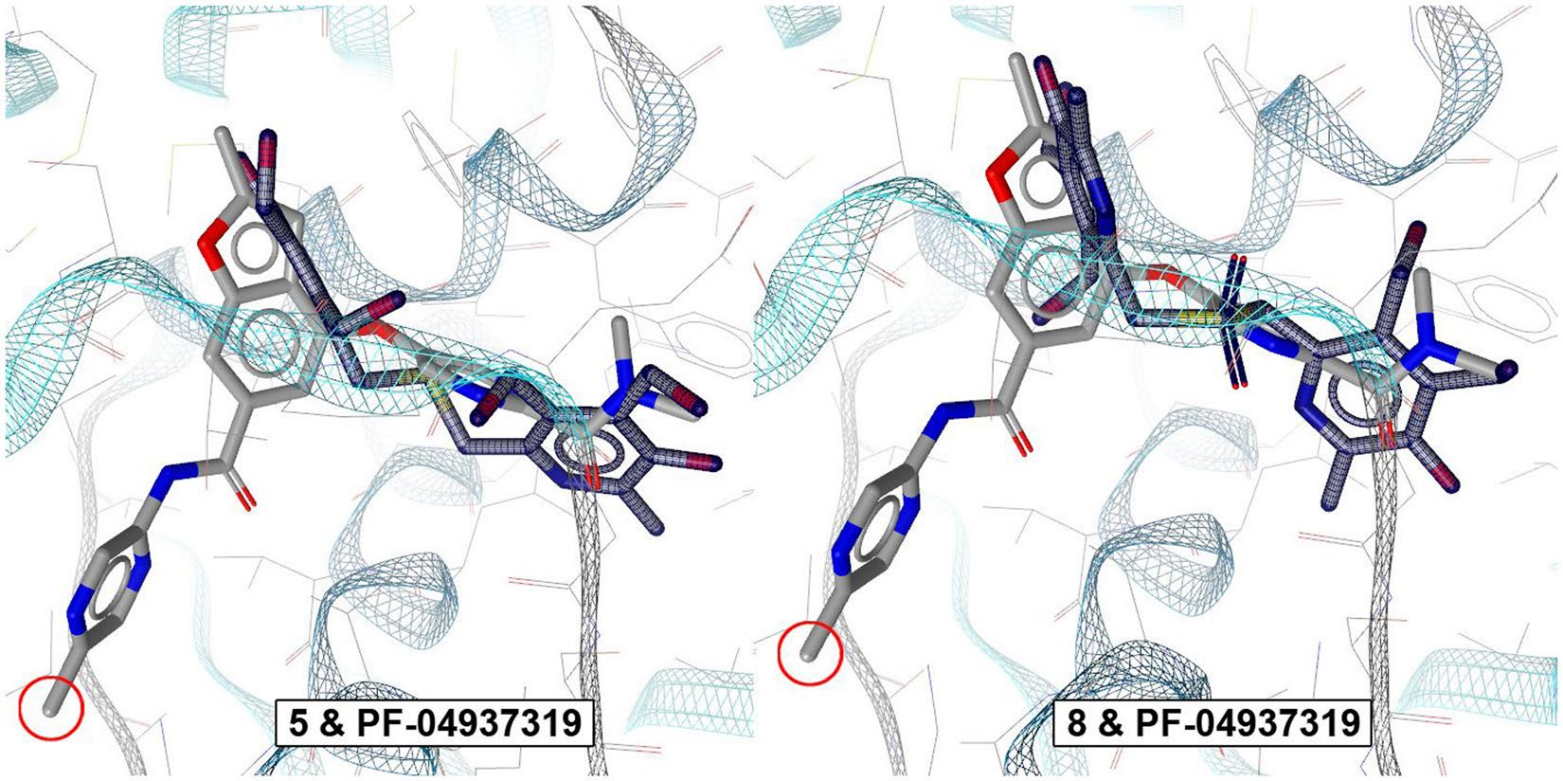
Comparison of the poses of molecules **5**, **8** and **PF-04937139** in the GK binding.

In molecule **5** there is a pyridine ring forming a stacking interaction with TYR214; three groups form hydrogen bonds with THR65, GLY68, TYR214; two fragments participate in non-specific hydrophobic interactions with VAL91, TRP99, VAL101, MET210, ILE211, TYR214, TYR215, MET235, LEU451, ALA454. In molecule **8** there is a pyridine ring forming a stacking interaction with TYR214; five groups form hydrogen bonds with THR65, GLU67, GLY68, SER69, TYR214; two fragments are involved in hydrophobic interactions with THR65, VAL91, TYR214, LEU451, ALA454, VAL455.

In molecule **PF-04937139** there is no stacking; only one group forms a hydrogen bond with ARG63; two fragments are involved in hydrophobic interactions with ILE159, MET210, ILE211, TYR214, MET235, and ALA456.

## Discussion

Comparison of the binding interactions demonstrates clear difference in the binding modes of the synthesized compounds and PF-04937139. Thus, the reference agent does not have any stacking interactions and forms only one hydrogen bond with amino acid ARG63 other than those involved in interaction with **5** and **8**. Of the six amino acids involved in hydrophobic interactions, the following ones are equivalent: MET210, ILE211, TYR214 and MET235 for PF-04937139 and **5**; TYR214 for PF-04937139 and **8**. It can be suggested that the hydrophobic interaction profiles of PF-04937139 and **5** are more similar than those of PF-04937139 and **8**. PF-04937139 has four binding points in the binding site, while compound 5 has seven binding points, and compound 8 has nine binding points. Thus, compounds 5 and 8 are more strongly and rigidly fixed to the binding site than PF-04937139. The following differences are observed in the calculated binding modes for structures 5 and 8 versus the reference agent PF-04937139: 1) a stacking with the pyridine ring; 2) increased number of hydrogen bonds; 3) two but not three areas of hydrophobic interactions; 4) the lack of a semi-rigid chain with an aromatic hydrophobic “tip”. These differences primarily suggest that the conformational adaptability of the activator molecule to the peculiarities of the allosteric site of glucokinase is essential for GK activity.

**Figure 5:**
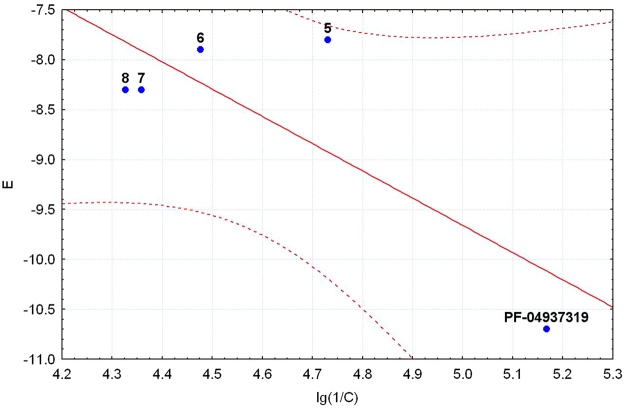
Comparison of GK activity and docking energy of compounds **5–8** and **PF-04937139**.

For comparison, Fig. 4 shows both molecules aligned with PF-04937139 in the binding site. The poses of **5** and **8** only partially overlapped with the pose of PF-04937139. In general, molecule **5** is better aligned with a N,N-dimethylpyrimidine-2-carboxamide moiety of PF-04937139 than molecule **8**. This difference can partially explain the higher *in vitro* activity of compound **5**. On the other hand, both compounds 5 and 8 do not have a second hydrophobic binding point with ALA456 and ILE159, which in molecule PF-04937139 is represented by a methyl group at position 5 of the pyrazine ring (marked in red in Fig. 4). In Fig. 5 we make a comparison between the GK activity and docking energy of compounds 5-8 and PF-04937139.

In conclusion, herein we report the synthesis, *in vitro* activity and cytotoxicity data, *in vivo* acute toxicity data, and the molecular docking study results for a series of novel potent activators of glucokinase. The leading compounds represent viable preclinical candidates for the treatment of type 2 diabetes mellitus, as well as a promising starting point for the design of structural analogs with improved activity.

## Methods

### Synthetic procedures

^1^H NMR spectra were recorded on Bruker AVANCE 400 spectrometer at operating frequency 400 MHz. ^13^C NMR spectra were recorded on a Bruker AVANCE 400 spectrometer at operating frequency 101.56 MHz. Chemical shifts were measured with reference to the residual protons of the solvents (DMSO-d_6_, ^1^H, 2.50 ppm, ^13^C, 39.52 ppm; CDCl_3_, ^1^H, 7.26 ppm, ^13^C, 77.16 ppm. Coupling constants (J) are given in Hertz (Hz). The following abbreviations are used to describe coupling: s = singlet; d = doublet). Melting points were determined using a Stanford Research Systems MPA-100 OptiMelt melting point apparatus and are uncorrected. For TLC analysis silica gel plates from Sorbfil (Krasnodar, Russia) were used with UV light (254 nm/365 nm) as developing agent. Column chromatography was performed on silica gel (60–200 mesh) from Acros.

HRMS mass spectra were obtained on a quadrupole time-of-flight (qTOF) AB Sciex Triple TOF 5600 mass spectrometer using turbo-ion spray source (nebulizer gas nitrogen, a positive ionization polarity, needle voltage 5500 V). Recording of the spectra was performed in a TOF MS mode with a collision energy 10 eV, declustering potential 100 eV and with resolution more than 30 000 full-width half-maximum. Samples with the analytes concentration 5 μmol/l were prepared by dissolving the test compounds in a mixture of methanol (HPLC-UV Grade, LabScan) and water (LC-MS Grade, Panreac) with a ratio of 1:1.

#### Bis((9-acetoxy-3,3,8-trimethyl-1,5-dihydro-[1,3]dioxepino[5,6-c]pyridin-6-yl)methyl)sulfide (3)

2.00 g (6.7 mmol) of compound **2** was dissolved in 50 ml of chloroform. 6.07 g (13.3 mmol) of sodium sulfide nonahydrate and 0.09 g (0.3 mmol) of tetrabutylammonium bromide were dissolved in 50 ml of distilled water. Obtained solutions were mixed together and vigorously stirred for 12 h at rt. Organic layer then was separated and evaporated in vacuo. The residue was purified using column chromatography (gradient of chloroform-ethyl acetate) to obtain 1.33 g (71%) of compound **3** as light yellow oil. ^1^H NMR (400 MHz, CDCl_3_) δ 1.49 (s, 12H), 2.34 (s, 6H), 2.35 (s, 6H), 4.61 (s, 4H), 4.71 (s, 4H), 4.98 (s, 4H). ^13^C NMR (100MHz, CDCl_3_) δ 19.04, 20.49, 23.72, 45.21, 59.05, 60.47, 102.82, 132.69, 141.22, 142.79, 149.23, 149.24, 168.20. HRМS-ESI: found [*М*+*Н*]^+^ 561.2265, C_28_H_36_N_2_O_8_S, calculated [*М*+*Н*]^+^ 561.2265.

#### Bis((9-acetoxy-3,3,8-trimethyl-1,5-dihydro-[1,3]dioxepino[5,6-c]pyridin-6-yl)methyl)disulfide (4)

3.37 g (14.0 mmol) of sodium sulfide nonahydrate was dissolved in 150 ml of distilled water and 0.45 g (14.1 mmol) of sulfur was added. The mixture was stirred until the complete dissolution of sulfur. After that 0.18 g (0.6 mmol) of tetrabutylammonium bromide was added. To the resulting mixture a solution of 4.21 g (14.0 mmol) of compound **2** in 100 ml of chloroform was added. Reaction mixture was vigorously stirred for 12 h at rt. Organic layer then was separated and evaporated in vacuo. The residue was purified using column chromatography (gradient of chloroform-ethyl acetate) to obtain 2.66 g (64%) of compound **4** as white crystalline solid with m.p. 141–143 °C. ^1^H NMR (400 MHz, DMSO-*d6*) δ 1.45 (s, 12Н); 2.30 (s, 6Н); 2.31 (s, 6Н); 3.93 (s, 4Н); 4.66 (s, 4Н); 4.90 (s, 4Н); ^13^С NMR (100 MHz, DMSO-*d6*) δ 18.98, 20.42, 23.68, 43.31, 58.88, 60.84, 102.63, 131.98, 140.63, 141.94, 149.07, 149.46, 168.21. HRМS-ESI: found [*М+Н*]^+^ 593.1986, C_28_H_37_N_2_O_8_S_2_, calculated [*М+Н*]^+^ 593.1986.

#### Bis(5-hydroxy-3,4-bis(hydroxymethyl)-6-methylpyridin-2-yl)methyl)sulfide dihydrochloride (5)

1.00 g (1.8 mmol) of compound **3** was dissolved in 20 ml of 2 M hydrochloric acid. Solution was stirred at 50 °C for 1 h. Then NaHCO_3_ was added until pH = 6.5. The precipitate formed was filtered off, washed with 10 ml of distilled water and dissolved in 10 ml of 2 M hydrochloric acid. This solution was evaporated in vacuo to obtain 0.74 g (89%) of compound **5** as white crystalline solid with m.p. 185–187 °С. ^1^H NMR (400 MHz, D_2_O) δ 2.56 (s, 6Н), 4.20 (s, 4Н), 4.73 (s, 4Н), 5.08 (s, 4Н); ^13^С NMR (100 MHz, D_2_O) δ 14.20, 30.08, 55.33, 57.02, 134.01, 140.64, 142.59, 143.59, 152.38. HRМS-ESI: found [*М–2Cl–Н*]^+^ 397.1428, C_18_H_26_Cl_2_N_2_O_6_S, calculated [*М–2Cl–Н*]^+^ 397.1428.

#### Bis(5-hydroxy-3,4-bis(hydroxymethyl)-6-methylpyridin-2-yl)methyl)disulfide dihydrochloride (6)

0.56 g (0.9 mmol) of compound **4** was dissolved in 20 ml of 2 M hydrochloric acid. Solution was stirred at 50 °C for 1 h. Then NaHCO_3_ was added until pH = 6.5. The precipitate formed was filtered off, washed with 10 ml of distilled water and dissolved in 10 ml of 2 M hydrochloric acid. This solution was evaporated in vacuo to obtain 0.38 g (85%) of compound **6** as white crystalline solid with m.p. 198–200 °С. ^1^H NMR (400 MHz, D_2_O) δ 2.61 (s, 6Н), 4.19 (s, 4Н), 4.74 (s, 4Н), 5.03 (s, 4Н); ^13^С NMR (100 MHz, D_2_O) δ 14.20, 35.54, 55.61, 57.21, 133.97, 140.69, 142.63, 143.36, 152.52. HRМS-ESI: found [*М–2Cl–Н*]^+^ 397.1428, C_18_H_26_Cl_2_N_2_O_6_S, calculated [*М–2Cl–Н*]^+^ 397.1428.

#### Bis(5-hydroxy-3,4-bis(hydroxymethyl)-6-methylpyridin-2-yl)methyl)sulfoxide dihydrochloride (7)

1.00 g (2.1 mmol) of compound **5** was suspended in 15 ml of glacial acetic acid and 0.35 ml (4.2 mmol) of 36% hydrogen peroxide solution in water was added. Distilled water was added dropwise to this mixture while stirring until the clear solution was obtained. Reaction mixture left overnight at r.t. and then evaporated in vacuum. Residue was dissolved in isopropanol, and upon standing the white precipitate was formed. The precipitate was filtered off and dried to obtain 0.87 g (84%) of compound **7** as white crystalline solid with m.p. 183–184 °C. ^1^H NMR (400 MHz, D_2_O) δ 2.62 (s, 6Н), 4.64 (d, *J* = 14.1 Hz, 2Н), 4.79 (s, 4Н), 4.89 (d, *J* = 14.1 Hz, 2Н), 5.05 (s, 4H); ^13^С NMR (100 MHz, D_2_O) δ 14.52, 51.40, 55.76, 57.09, 133.88, 136.02, 141.91, 144.98, 153.27. HRМS-ESI: found [*М–2Cl–Н*]^+^ 413.1377, C_18_H_26_Cl_2_N_2_O_7_S, calculated [*М–2Cl–Н*]^+^ 413.1377.

#### Bis(5-hydroxy-3,4-bis(hydroxymethyl)-6-methylpyridin-2-yl)methyl)sulfone dihydrochloride (8)

1.00 g (2.1 mmol) of compound **5** was suspended in 15 ml of glacial acetic acid and 1.43 ml (17.0 mmol) of 36% hydrogen peroxide solution in water was added. Distilled water was added dropwise to this mixture while stirring until the clear solution was obtained. Reaction mixture was stirred for 4 h at 50 °C and then evaporated in vacuo. Residue was dissolved in propanol, and upon standing the white precipitate was formed. The precipitate was filtered off and dried to obtain 0.55 g (52%) of compound **8** as white crystalline solid with m.p. 181–183 °C. ^1^H NMR (400 MHz, DMSO-*d*_*6*_) δ 2.60. (s, 6Н), 4.68 (s, 4Н), 4.90 (s, 4Н), 5.30 (s, 4Н); ^13^С NMR (100 MHz, DMSO-*d*_*6*_) δ 15.96, 54.09, 55.72, 56.09, 131.94, 137.86, 140.92, 144.57, 152.54. HRМS-ESI: found [*М–2Cl–Н*]^+^ 429.1326, C_18_H_26_Cl_2_N_2_O_8_S, calculated [М–2Cl–Н]^+^ 429.1326.

### Molecular modeling study

The building of 10 conformations of each compound was performed in the program MarvinSketch 17.1.23 (ChemAxon Kft. http://www.chemaxon.com/products/marvin/marvinsketch/). These conformations were optimized in the MOPAC2016 program (Stewart Computational Chemistry, http://openmopac.net) and the best ones with minimal energy were selected.

Docking the best conformations of molecules **5–8** and **PF-04937139** in three X-ray models of the human GK (PDB codes are 3H1V, 4ISE, 4IXC) was carried out using the program AutoDock Vina 1.1.1^18^, every compound 5 times per model, with the determination among 15 calculated values of the minimum docking energy. Three best models were selected from the 30 X-ray models by means of the procedure outlined in^17^. 35 known GKAs were selected, passing through the 1st, 2nd or 3rd stages of clinical trials. The conformations of these 35 standards were optimized using the procedure described above. The best conformations of the standards (five times in each model) were docked in 30 X-ray GK models found in the PDB https://www.rcsb.org/. For each GK model, the average value of the minimum docking energies of the standards was calculated. The three best models were selected in accordance with three minimum average values of docking energies of standards.

The location of the allosteric site was determined using information about the binding amino acids according to the data previously published^19^. Two lists of binding amino acids were compared: one was determined by the method of point mutations^20^ and second was found by X-ray analysis^19^. As a result, the list of 19 key amino acids of the GK site was obtained: Val62, Arg63, Ser64, Thr65, Gly68, Ser69, Gly72, Val91, Trp99, Met210, Ile211, Tyr214, Tyr215, Met235, Leu451, Val452, Val455, Lys458, Lys459. Based on this data, using the PyRx 0.8 program https://sourceforge.net/projects/pyrx/, where the docking space covering the allosteric GK site was built. Analysis of the binding mechanism was performed using the LigandScout 4.1 program (Ligand GmbH. http://www.inteligand.com/ligandscout/).

### Cytotoxic activity

*In vitro* experiments were carried out in accordance with relevant guidelines and regulations set forth by the European Communities Council Directive 2010/63/EU. All experimental protocols were approved by the Animal Care and Use Committee of Kazan Federal University, Russia and informed consent was obtained from all subjects. Briefly, human skin fibroblasts (HSFs) were isolated from the skin explant according to the conventional protocol^21^. HSFs cells were cultured in the minimum essential medium Eagle (α-MEM) supplemented with 10% fetal bovine serum, 2 mM L-glutamine, 100 µg/mL streptomycin and 100 U/mL penicillin under standard conditions (37 °C, 5% CO_2_ atmosphere). Adhered cells were collected from the culture flask by detaching them with trypsin-EDTA solution. Suspended cells were washed by centrifugation at 200 g in PBS.

Cytotoxic concentrations (CC_50_) of compounds were determined with the use of MTT assay. Cells were pre-seeded in 96-well plate at the density of 2000 cells per well and cultured with adding a series of diluted water solutions of compounds for 3 days under standard conditions. Culture medium in the plate was then replaced by the fresh one supplemented with 20 μL 5 mg/mL MTT and additionally kept for 3 h to allow for reduction of MTT into colored product (formazan) by metabolically active cells. Then the solution was removed and DMSO (100 μL) was added to solubilize the formazan crystals. Optical absorbance of produced formazan, proportional to viable cell number, was registered on Infinite 200 PRO analyzer at 550 nm (the reference wavelength 650 nm). CC_50_ values were generated by fitting relative responses to the mean of mQ treated controls using the variable slope dose-response curve fitting function within Origin software. Upper bounds for curve fitting were set as the mean of mQ treated negative controls. At least nine dose response points and three biological replicates were used to determine dose response curves.

### Acute toxicity in rats

*In vivo* experiments were carried out in accordance with relevant guidelines and regulations set forth by the European Communities Council Directive 2010/63/EU. All experimental protocols were approved by the Animal Care and Use Committee of Kazan Federal University, Russia. Toxicological experiments were performed using intragastric injection of the different compounds in rats weighting 180–220 g. Rats were maintained on a 12 h light/dark cycle (light from 7:00 a.m. to 7:00 p.m.) at 20–22 °C and 60–70% relative humidity. One dose of 2000 mg/kg (causing compound is likely to be nontoxic) was used with 12 animals (6 males, 6 females). Animals are observed individually after dosing at least once during the first 30 minutes, periodically during the first 24 hours, with special attention given during the first 4 hours, and daily thereafter, for a total of 14 days. During these period symptoms of intoxication were recorded. LD_50_, dose (in mg/kg) causing lethal effects in 50% of animals, was taken as a criterion of toxicity.

## Electronic supplementary material

Supplementary information.
