# Author Correction: Pyridoxine dipharmacophore derivatives as potent glucokinase activators for the treatment of type 2 diabetes mellitus

**DOI:** 10.1038/s41598-018-24838-6

**Published:** 2018-04-19

**Authors:** Mikhail S. Dzyurkevich, Denis A. Babkov, Nikita V. Shtyrlin, Olga Yu. Mayka, Alfiya G. Iksanova, Pavel M. Vassiliev, Konstantin V. Balakin, Alexander A. Spasov, Vadim V. Tarasov, George Barreto, Yurii G. Shtyrlin, Gjumrakch Aliev

**Affiliations:** 1 Kazan (Volga region) Federal University, Kremlyovskaya 18, Kazan, 420008 Russia; 2 Volgograd State Medical University, Pavshikh Bortsov Sq. 1, Volgograd, 400131 Russia; 3 I.M. Sechenov First Moscow State Medical University, Trubetskaya St. 8, bld 2, Moscow, 119991 Russia; 4 Institute of Pharmacy and Translational Medicine, Sechenov First Moscow State Medical University, 119991 Moscow, Russia; 5 Departamento de Nutrición y Bioquímica, Facultad de Ciencias, Pontificia Universidad Javeriana, Bogotá, D.C. Colombia; 6 Instituto de Ciencias Biomédicas, Universidad Autónoma de Chile, Santiago, Chile; 7 GALLY International Biomedical Research & Consulting LLC 7733 Louis Pasteur Dr. Suite #328, San Antonio, TX 78229 USA; 8 School of Health Science and Healthcare Administration, University of Atlanta, E. Johns Crossing, #175, Johns Creek, GA 30097 USA; 9 Institute of Physiologically Active Compounds Russian Academy of Sciences, Chernogolovka, 142432 Russia

Correction to: *Scientific Reports* 10.1038/s41598-017-16405-2, published online 22 November 2017

In this Article, an earlier version of Figure 1 is shown. The correct version of Figure 1 appears below.

**Figure 1:**
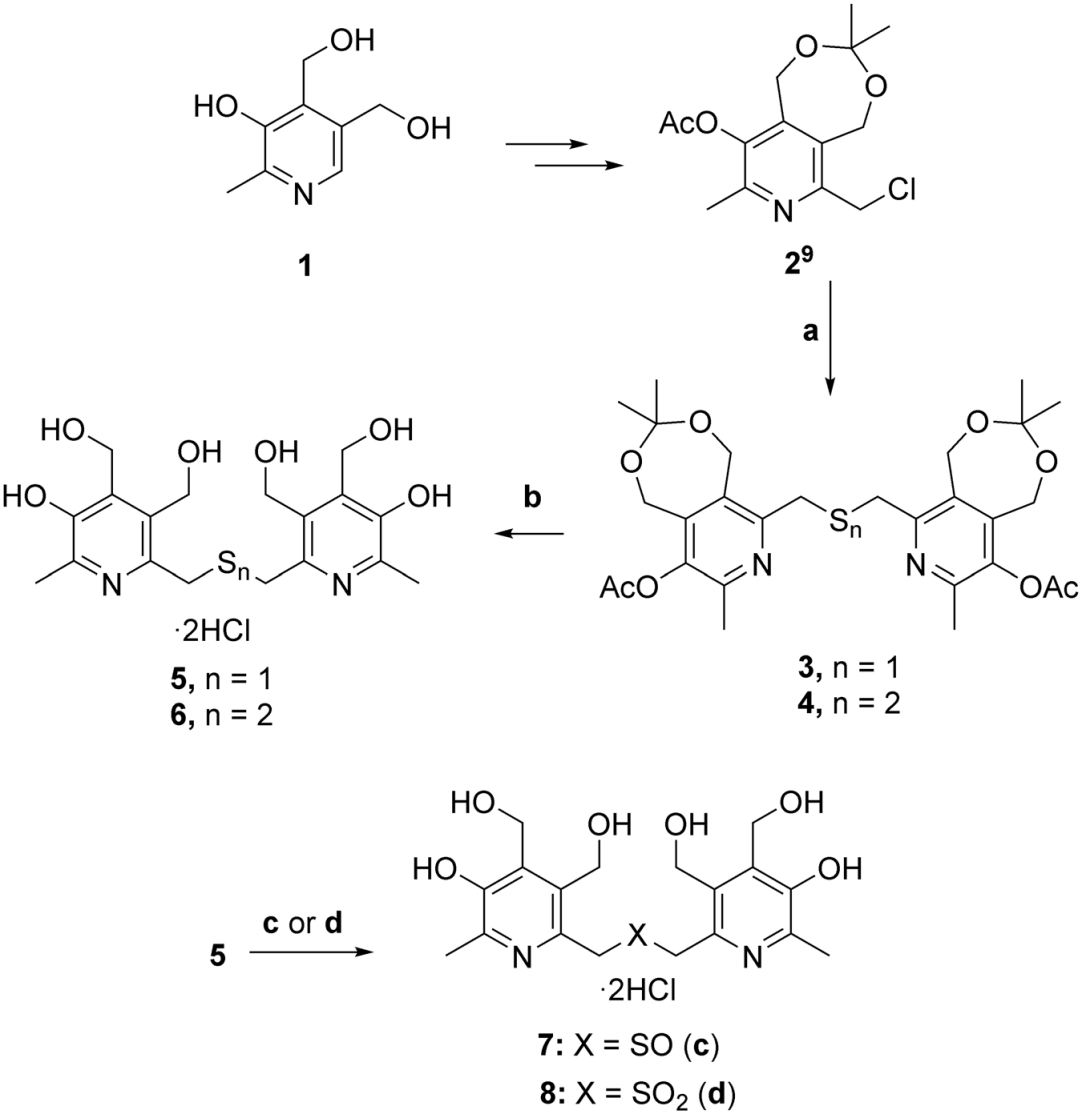
Synthetic route of compounds used. (**a**) Na2Sx, TBAB, H2O-CHCl3, rt, 12 h; (**b**) H2O, HCl, 40 °C, 3 h; (**c**) H2O2, AcOH/H2O, rt, 4 h; (**d**) H2O2, AcOH/H2O, 50 °C, 4 h.

