# Supplementary material for: Pyridoxine dipharmacophore derivatives as potent glucokinase activators for the treatment of type 2 diabetes mellitus
Source: Sci Rep. 2017 Nov 22;7:16072. doi: 10.1038/s41598-017-16405-2 (PMC5700121; doi:10.1038/s41598-017-16405-2)
Supplement: Supplementary file 1 — Supplementary information. [file 41598_2017_16405_MOESM1_ESM.doc]

**Pyridoxine dipharmacophore derivatives as potent glucokinase activators for the treatment of type 2 diabetes mellitus**

Mikhail S. Dzyurkevich ^a^, Denis A. Babkov ^b^, Nikita V. Shtyrlin ^a^, Olga Yu. Mayka ^b^, Alfiya G. Iksanova ^a^, Pavel M. Vassiliev ^b^, Konstantin V. Balakin ^a,c^, Alexander A. Spasov ^b^, Vadim V. Tarasov ^d^, George Barreto ^e,f^, Yurii G. Shtyrlin^a^, Gjumrakch Aliev^g,h,*^

^a^ Kazan (Volga region) Federal University, Kremlyovskaya 18, Kazan, 420008, Russia.

^b^ Volgograd State Medical University, Pavshikh Bortsov Sq. 1, Volgograd, 400131, Russia.

^c^ I.M. Sechenov First Moscow State Medical University, Trubetskaya St. 8, bld 2, Moscow, 119991, Russia.

^d^ Institute of Pharmacy and Translational Medicine, Sechenov First Moscow State Medical University, 119991 Moscow, Russia.

^e^ Departamento de Nutrición y Bioquímica, Facultad de Ciencias, Pontificia Universidad Javeriana, Bogotá D.C., Colombia.

^f^ Instituto de Ciencias Biomédicas, Universidad Autónoma de Chile, Santiago, Chile.

^g^ GALLY International Biomedical Research & Consulting LLC 7733 Louis Pasteur Dr. Suite #328, San Antonio, TX, 78229 USA.

^h^ School of Health Science and Healthcare Administration, University of Atlanta, E. Johns Crossing, #175, Johns Creek, GA, 30097 USA.

*Corresponding author, e-mail: [aliev03@gmail.com](mailto:aliev03@gmail.com)

**Suppl. Fig. 1.** GK activators which entered phase II clinical trials.
